# Factors Associated With Sputum Culture-Negative vs Culture-Positive Diagnosis of Pulmonary Tuberculosis

**DOI:** 10.1001/jamanetworkopen.2018.7617

**Published:** 2019-02-08

**Authors:** Minh-Vu H. Nguyen, Natalie S. Levy, Shama D. Ahuja, Lisa Trieu, Douglas C. Proops, Jacqueline M. Achkar

**Affiliations:** 1 Department of Internal Medicine, University of California, Davis Health, Sacramento; 2 Department of Medicine, Albert Einstein College of Medicine, Bronx, New York; 3 Bureau of Tuberculosis Control, New York City Department of Health and Mental Hygiene, Queens, New York; 4 Department of Microbiology and Immunology, Albert Einstein College of Medicine, Bronx, New York

## Abstract

**Importance:**

Recognition of active tuberculosis (TB) in its earliest stages could reduce morbidity and prevent advancement to transmissible disease. Little is published about the occurrence and presentation of sputum culture-negative pulmonary TB (PTB), an early paucibacillary but often underrecognized disease state.

**Objective:**

To assess differences between culture-negative and culture-positive PTB regarding occurrence, clinical presentation, radiographic findings, demographics, and comorbidities.

**Design, Setting, and Participants:**

Cross-sectional study in which surveillance data of adult patients with PTB reported to the New York City Department of Health in New York, New York, from 2011 through 2013, ie, years for which demographic, clinical, and radiographic data were collected. Patients were aged 18 years or older, had signs of pulmonary disease, and had mycobacterial sputum culture results; those with HIV coinfection or a TB diagnosis within 2 years prior to presentation were excluded. Culture-negative PTB was defined as clinical and radiographic presentation consistent with TB, 3 negative results on sputum culture, and improvement with antituberculous treatment. The analyses were performed between 2015 and 2016; notably, the proportion of reported patients with culture-negative PTB has remained consistent during the past 2 decades.

**Main Outcomes and Measures:**

The occurrence of culture-negative PTB among all patients with PTB was calculated, and demographics, comorbidities, symptoms, and radiographic findings were compared between culture-negative and culture-positive PTB.

**Results:**

Of the 796 patients with PTB (median [interquartile range] age, 41 [29-54] years; 499 [63%] men) who met criteria for analysis, 116 (15%) had negative results on sputum culture. Patients with culture-negative PTB compared with culture-positive PTB were less frequently male (53% vs 64%; *P* = .03) and presented with a significantly lower frequency of cough (68% vs 89%; *P* < .001), weight loss (39% vs 51%; *P* = .03), and cavitation on both chest radiograph (7% vs 28%; *P* < .001) and chest computed tomographic scan (26% vs 59%; *P* < .001).

**Conclusions and Relevance:**

Given the lack of criterion-standard test confirmation and the relative paucity of symptoms and radiological abnormalities, culture-negative PTB is likely underdiagnosed and its occurrence underestimated globally. Awareness of these findings, enhanced diagnostic approaches, and, ideally, better biomarkers could improve detection and treatment of this early disease and reduce the development of transmissible TB.

## Key Points

**Question:**

What proportion of patients with pulmonary tuberculosis have negative sputum culture results, and how do they present differently from patients with culture-positive pulmonary tuberculosis?

**Findings:**

In our cross-sectional study of 796 patients without HIV infection who were diagnosed with pulmonary tuberculosis, sputum culture-negative pulmonary tuberculosis represented 15% of all adult patients with pulmonary tuberculosis in New York, New York. These patients had a significantly lower proportion of cough, weight loss, any symptom in general, and cavitation on imaging compared with patients with sputum culture-positive disease.

**Meaning:**

Through awareness of these findings, the detection and treatment of this likely early disease could potentially be improved and the development of transmissible tuberculosis reduced.

## Teaser

This cross-sectional study compares the occurrence, clinical presentation, radiographic findings, demographics, and comorbidities in adult patients with culture-negative vs culture-positive pulmonary tuberculosis.

## Introduction

Active tuberculosis (TB) is a global public health problem and one of the world’s leading causes of mortality. In 2017, 10.4 million individuals were newly diagnosed with TB globally, with 1.6 million associated deaths.^1^ Early detection and treatment of TB reduce the transmission of *Mycobacterium tuberculosis* complex (*M tuberculosis*). Diagnosis is typically confirmed by detection of *M tuberculosis* in culture or nucleic acid amplification (NAA) testing.^2,3^ In areas with sufficient resources and high levels of experience with the range of clinical presentations of TB, approximately 20% to 30% of patients with TB are found to lack culture confirmation and are diagnosed clinically.^4,5,6,7^

Culture-negative pulmonary TB (PTB) in patients without HIV coinfection is likely an early disease state on the continuum between *M tuberculosis* infection and disease, which, if left untreated, can advance to culture-positive disease.^8,9,10,11^ Examining and outlining the clinical presentation of culture-negative PTB could increase clinicians’ awareness, facilitate the recognition of PTB at an early state, and lead to early treatment initiation, which in turn could reduce the development of transmissible disease. Furthermore, because treatment of culture-negative PTB is shorter than that of culture-positive PTB (4 vs 6 months, respectively),^12,13,14^ individuals may have fewer medication adverse effects and may be less likely to relapse, owing to increased adherence and treatment completion.

The occurrence and clinical presentations of culture-negative PTB have been insufficiently studied. Of the few studies including culture-negative disease, a Hong Kong study in 1981 found that culture-negative PTB was associated with less hemoptysis and radiographic abnormalities than acid-fast bacilli smear–negative, culture-positive PTB.^9^ In a recent pilot study of patients without HIV infection who had PTB, we found that patients with culture-negative compared with culture-positive PTB had less cough, sputum production, weight loss, and cavitary lesions on chest computed tomography (CT), suggesting that classic TB symptoms are not always associated with culture-negative disease.^4^ However, population-based studies are needed to investigate the occurrence and validate the clinical presentations of culture-negative TB.

We investigated the clinical presentation of patients diagnosed with culture-negative PTB in New York City, New York (NYC), using surveillance data from the NYC Department of Health on patients with verified TB from 2011 through 2013. Owing to its large immigrant population, NYC is one of the few metropoles in the United States with a higher incidence of TB (7.5 cases per 100 000 population) than the US national average (2.8 cases per 100 000 population).^6,15^ In 2017, NYC had an increase of 10% in patients with TB (the largest increase since 1992), raising major public health concerns.^6^ For the past 2 decades, approximately 17% of all patients with TB in NYC have been reported to have culture-negative results, but the specific proportion of culture-negative pulmonary disease in not overtly immunocompromised individuals has, to our knowledge, neither been reported nor studied with regard to its clinical presentation.^6^ Because of the differences in pathogenesis and clinical presentation in the presence of considerable immunosuppression,^8,11^ we focused our investigation on patients without evidence of HIV coinfection. Based on our pilot data, we hypothesized that patients with culture-negative PTB present with fewer symptoms and fewer radiographic abnormalities compared with patients with culture-positive PTB. Our primary objectives were to evaluate the differences between patients with culture-negative and culture-positive PTB with regard to their occurrence, the frequency and duration of TB-associated symptoms, and the frequency of cavitary lesions on chest radiograph (CXR) and chest CT. Our secondary objectives were to evaluate potential differences between the groups with regard to demographics and comorbidities.

## Methods

### Reporting of Suspected TB Cases and Counting Cases in NYC

The NYC Health Code requires clinicians to report individuals with suspected or diagnosed TB within 24 hours to the health department.^16^ Once reported, a health department case manager is assigned to follow the individual, most commonly until treatment completion or until the clinician determines that the individual does not have TB.^14^ Persons may be verified to have TB based on either laboratory finding (culture or NAA assay) or on a clinical basis (improvement of symptoms or imaging abnormalities following antituberculous treatment).

### Study Design, Setting, and Population

In this cross-sectional study, we reviewed the demographic and clinical data for all verified patients with TB in NYC from January 1, 2011, through December 31, 2013. Inclusion criteria were being aged 18 years or older; having a pulmonary site of disease; having at least 1 positive mycobacteriology sputum culture result, or, if negative, at least 3 sputum culture results; and having initiated TB treatment. Exclusion criteria were having known HIV infection; not being alive when reported; and having no record of treatment initiation date. We also excluded those who were diagnosed within 9 months of known exposure to a patient with TB because these were actively followed by the health department and, as such, differed from our study population; were diagnosed with TB within 2 years prior to presentation because of their potentially high likelihood for TB relapse^17^; lacked imaging results in the absence of microbiological confirmation; or had positive culture results for nontuberculous mycobacteria, to reduce the possibility of misdiagnosis bias.

We conducted this study in accordance with the amended Declaration of Helsinki.^18^ The institutional review boards of the Albert Einstein College of Medicine and the NYC health department deemed the study exempt from review and waived the requirement for informed consent because the data were derived from a surveillance data set with deidentified data. We used the Strengthening the Reporting of Observational Studies in Epidemiology (STROBE) reporting guideline for cross-sectional studies to conduct this study.^19^

### Measurements, Diagnoses, and Definitions

A patient’s culture status was defined based on specimens collected during the 8 weeks before initiation of TB treatment. A PTB diagnosis was defined as clinical and/or radiographic and/or microbiological evidence of pulmonary disease resulting in treatment initiation by the diagnosing clinician. Culture-positive PTB was defined as having at least 1 positive culture from a sputum specimen collected in the 8 weeks prior to treatment initiation. The American Thoracic Society (ATS) and Centers for Disease Control and Prevention (CDC) recommend collection of 3 sputum samples for acid-fast bacilli smear and mycobacterial cultures.^20^ Consistent with these recommendations and following the diagnostic guidelines, we defined patients as having culture-negative PTB if they had 3 initial sputum cultures negative for *M tuberculosis* plus met the following inclusion criteria: (1) presented with signs and/or symptoms consistent with PTB combined with being reported by the diagnosing clinician to the NYC health department; and (2) had documented clinical or radiographic improvement on antituberculous treatment by a health department case manager who followed the patients during the period of antituberculous treatment.^20^ Requiring a chest CT and/or bronchoalveolar lavage result as an inclusion criterion for culture-negative PTB was not feasible because not all patients received these diagnostic modalities, which, although encouraged if clinically indicated, are not recommended as standard approach in the ATS and CDC diagnostic guidelines.^20^ Culture-negative PTB included an *M tuberculosis*–positive result by culture or NAA assay from a specimen other than the initial 3 sputum samples to maintain consistent criteria for the entire study population (many patients received only 3 sputum cultures as per diagnostic guidelines). A subgroup analysis was done on patients with culture-negative PTB who received sputum cultures beyond the initial 3 and ultimately had a positive result.

We compared demographic and clinical characteristics as well as symptoms and cavitary lesions on CXR or chest CT between the groups. Demographic characteristics assessed included age at report, sex, and foreign birth, which we defined as birth outside the United States or a US territory. Comorbidities assessed were presence of diabetes, end-stage renal disease, and any malignant neoplasm. Overall, symptoms were coded as present, not present, unknown, or missing, and they included the presence and duration of cough, sputum production, fever, night sweats, involuntary weight loss, hemoptysis, swollen glands, and chest pain. The presence of cavitary lesions was assessed based on the initial CXR or, if available, chest CT results.

### Statistical Analysis

We obtained the data set from the NYC health department electronic surveillance and case management system, Maven (Consilience Software) and performed statistical analysis using Stata software version 13.1 IC (StataCorp). A 2-tailed 5% significance level was used for all analyses. Continuous variables were assessed for normality and homoscedasticity and analyzed by either the *t* test or Mann-Whitney *U* test. Categorical variables were analyzed using the Pearson χ^2^ test without correction for continuity or the Fisher exact test (for expected counts <5). We corrected for missing data by either removing observations from analysis or coding them as not present. We used this approach because of the limitation inherent to surveillance data and that not all clinicians specify lack of particular symptoms in their documentation.

## Results

From January 2011 through December 2013, 1799 adults were diagnosed with active TB in NYC. Among these, 796 (44%) met criteria for analysis (Figure). The median age of the 796 patients analyzed was 41 years (interquartile range, 29-54 years) and 499 (63%) were men. Based on their initial 3 sputum cultures, 116 patients (15%) were categorized as having culture-negative results. Of note, 26 patients with culture-negative PTB had a positive *M tuberculosis* culture or NAA result in a specimen collected beyond the initial 3 sputum samples, including 11 with a subsequent positive sputum culture result (the other 15 patients had a subsequent positive sputum NAA result but continuous negative sputum culture results for *M tuberculosis*). As described in the Methods section, these 11 patients were counted as having culture-negative PTB with an additional subanalysis to assess differences between the groups if they were counted as culture-positive cases.

**Figure.**
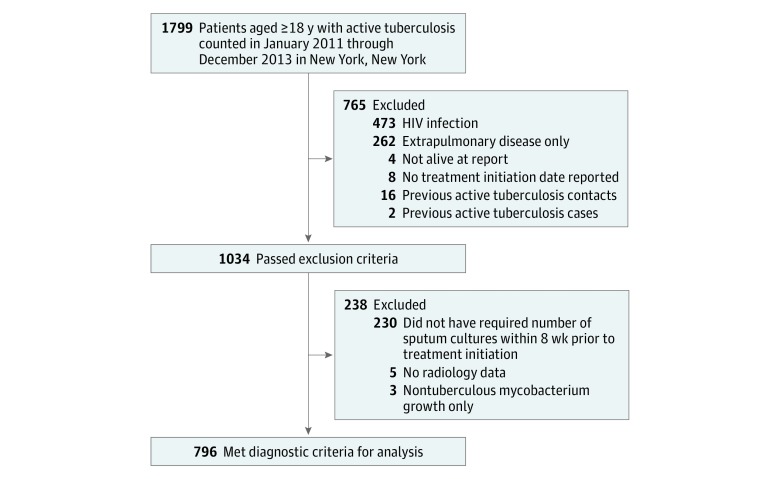
Flowchart of Study Population

### Demographics and Comorbidities

Patients with culture-negative PTB were significantly less likely to be men when compared with patients with culture-positive PTB (53% vs 64%; *P* = .03). There were no other significant differences in demographics between the 2 groups (Table 1).

**Table 1.** Demographics and Comorbidities of Patients With Active PTB

Variable^a^	PTB Culture Result, No. (%)		*P* Value^b^
	Negative (n = 116)	Positive (n = 680)	
Age, median (IQR), y	42 (32-56)	40 (29-54)	.37
Male	62 (53)	437 (64)	.03
Foreign born	104 (90)	601 (88)	.69
Diabetes	14 (12)	119 (18)	.15
Any renal disease	3 (3)	7 (1)	.17
End-stage renal disease	1 (1)	3 (0.4)	.47
Requires hemodialysis	1 (1)	2 (0.3)	.38
Any cancer	6 (5)	18 (3)	.14

Abbreviations: IQR, interquartile range; PTB, pulmonary tuberculosis.

^a^ Comorbidities are categorized as no or unknown vs yes.

^b^ Calculated based on the Pearson χ^2^ test without correction for continuity or the Fisher exact test (for expected counts <5).

### Clinical and Radiographic Presentation

A significantly lower proportion of patients with culture-negative PTB, compared with patients with culture-positive PTB, reported cough (68% vs 89%; *P* < .001), weight loss (39% vs 51%; *P* = .03), or any symptom in general (74% vs 90%; *P* < .001) (Table 2). Patients with culture-negative PTB were significantly less likely than those with culture-positive disease to have cavities on either CXR (7% vs 28%; *P* < .001) or CT (26% vs 59%; *P* < .001). In both groups, cavities were more frequently found on chest CT than on CXR (Table 2). We observed a significant positive trend for cough from patients with culture-negative PTB (67%) to those with culture-negative PTB with positive culture results beyond the initial 3 sputum samples (73%) to patients with culture-positive PTB (89%) in a 3-group comparison (*P* for trend <.001). When the patients with culture-negative PTB with positive culture results beyond the initial 3 sputum samples were combined with those with culture-positive instead of culture-negative disease for analysis, statistical significance for the results shown in Table 2 did not change. When we coded missing data as not present, patients with culture-negative PTB also had a significantly lower frequency of sputum production (31% vs 53%; *P* < .001), fever (35% vs 46%; *P* = .03), night sweats (22% vs 34%; *P* = .01), and hemoptysis (13% vs 23%; *P* = .02) than those with culture-positive PTB (Table 3).

**Table 2.** Clinical and Radiographic Presentation of Patients With Active PTB Stratified by Sputum Culture Results

Symptom or Finding	PTB Culture Result, No. (%)^a^		*P* Value^a^^,^^b^
	Negative (n = 116)	Positive (n = 680)	
Cough	57 (68)	543 (89)	<.001
Sputum production	36 (64)	366 (68)	.58
Fever	41 (49)	313 (51)	.66
Night sweats	25 (30)	230 (38)	.16
Weight loss	40 (39)	317 (51)	.03
Hemoptysis	15 (18)	155 (25)	.12
Swollen glands	7 (8)	22 (4)	.07
Chest pain	28 (33)	190 (31)	.71
No symptoms	30 (26)	68 (10)	<.001
AFB smear positive	0	376 (63)	<.001
Cavitation on CXR	10 (7)	186 (28)^c^	<.001
Cavitation on chest CT	22 (26)^d^	243 (59)^d^	<.001

Abbreviations: AFB, acid-fast bacilli; CT, computed tomography scan; CXR, chest radiograph; PTB, pulmonary tuberculosis.

^a^ Missing data were removed from analyses. Numbers of patients with data available were as follows: cough, 695 patients (13% missing); sputum production, 595 patients (25% missing); fever, 693 patients (13% missing); night sweats, 694 patients (13% missing); weight loss, 729 patients (8% missing); hemoptysis, 694 patients (13% missing); swollen glands, 692 patients (13% missing); chest pain, 693 patients (13% missing); AFB smear positivity, 707 patients (11% missing); cavitation on CXR, 789 patients (1% missing); and cavitation on chest CT, 482 patients (39% missing).

^b^ Calculated based on the Pearson χ^2^ test without correction for continuity or the Fisher exact test (for expected counts <5).

^c^ All 7 patients with missing CXR data had culture-positive results and thus were included in the patients analyzed.

^d^ Chest CT was performed in 73 of 116 patients (63%) with sputum culture-negative PTB and 416 of 680 patients (61%) with culture-positive PTB.

**Table 3.** Symptoms of Patients With Active PTB With Missing Data Coded as Not Present

Symptom	PTB Culture Result, No. (%)		*P* Value^a^
	Negative (n = 116)	Positive (n = 680)	
Cough	57 (49)	543 (80)	<.001
Sputum production	36 (31)	366 (53)	<.001
Fever	41 (35)	313 (46)	.03
Night sweats	25 (22)	230 (34)	.01
Weight loss	40 (34)	317 (47)	.02
Hemoptysis	15 (13)	155 (23)	.02
Swollen glands	7 (6)	22 (3)	.17
Chest pain	28 (24)	190 (28)	.43

Abbreviation: PTB, pulmonary tuberculosis.

^a^ Calculated based on the Pearson χ^2^ test without correction for continuity or the Fisher exact test (for expected counts <5).

Symptom duration was recorded by the health department only for cough, productive cough, and hemoptysis. Of these, cough (including the duration of cough) had the least missing data, with information available for 583 of 796 patients (73%). Our analysis with these data shows that patients with sputum culture-negative PTB reported a median (interquartile range) of 30 (12.5-65) days of cough compared with 34 (17-78) days among those with sputum culture-positive PTB (*P* = .22). However, because only 57 of 116 patients with culture-negative PTB (49%) were reported as having cough in contrast to 543 of 680 patients with culture-positive PTB (80%) when missing symptom information was coded as not present (Table 3), the comparison for the duration of cough has to be interpreted with caution.

## Discussion

Results from this NYC population-based study are consistent with data from our prior small pilot study.^4^ They highlight that patients without HIV infection who had culture-negative PTB presented with fewer symptoms and were less likely to have cavitary lesions than patients with culture-positive PTB. The observed attenuated symptoms and lower proportion of cavitation in culture-negative PTB are consistent with early paucibacillary disease. Although culture-negative PTB could include multiple disease states, we propose that most patients have disease that lies between incipient and active culture-positive PTB on the continuum of this complex illness.^8,11^ The trend of increasing cough associated with the transition of culture-negative to culture-positive PTB further supports this notion. With its relative paucity of clinical manifestations, this early disease state, although known to TB-experienced clinicians, might be underrecognized by clinicians who do not commonly see and diagnose TB.

Combined with a thorough exclusion of other potential diagnoses, recognition of and prompt treatment initiation for culture-negative PTB are important because of the high likelihood of progression to transmissible culture-positive disease if left untreated.^8,9,10^ This is supported by studies from Hong Kong and South Africa in the pre-HIV era showing that 40% and 58% of patients with culture-negative TB, respectively, progressed to developing culture-positive disease; if left untreated, these patients presented within 3 months to 65 months not just with culture conversion, but also with increased morbidity and extended pulmonary damage on imaging.^9,10^ An increased awareness of the characteristics of this early paucibacillary disease stage should prompt a more thorough evaluation, such as with a chest CT scan or consultation with a TB specialist, and should improve timely treatment initiation. Our findings further emphasize the continuous need for better biomarkers to help detect early TB.

Prompt recognition and timely treatment initiation of TB are cornerstones to its control globally. In addition to the importance in TB endemic regions, early recognition and treatment could also have an impact at the local levels in cities with high proportions of foreign-born inhabitants such as London, England, and NYC, especially in light of NYC’s recent 10% increase in TB incidence—the largest since 1992.^6^ The percentage of patients with culture-negative PTB in NYC constituted 15%, similar to the 13% reported for England in 2017 with the majority of cases from London.^7^ Of note, the proportion of culture-negative TB found in our pilot study was 21%, higher than that of the current study.^4^ In both of our studies, the definition was based on the same criteria: results of the initial 3 sputum cultures, the frequency of sputum sample analysis recommended by the ATS and CDC.^20^ A possible explanation for this difference is that our pilot study was conducted in public hospitals with higher annual TB case rates than many other NYC hospitals. Clinicians in these hospitals might thus have been more experienced with recognizing and comfortable with treating early TB disease states even in the absence of culture confirmation.

Postmortem studies in the pre-HIV era and in settings with varying TB burden have shown that it was common for clinicians to underrecognize TB.^21,22,23,24^ In this study, patients with culture-negative PTB were less likely to have cough, weight loss, and pulmonary cavitation, consistent with prior, more limited studies.^4,9^ The list of differential diagnoses in these patients can be broad, ranging from other slowly progressing infectious diseases such as histoplasmosis to autoimmune diseases such as sarcoidosis, making its diagnosis even more challenging. It is thus conceivable that clinicians, especially those who do not frequently see patients with TB, may refrain from consulting a TB specialist or starting antituberculous treatment in the absence of culture or biopsy confirmation and diminished radiographic findings and hallmark symptoms of cavitary TB. The caveat of this deferral is that these patients are often lost to follow-up until they become more symptomatic and develop culture-positive disease.

An early TB diagnosis has considerable advantages for patients and their communities. In addition to reducing transmission by treating a disease before it becomes infectious, treating noncavitary TB disease may result in higher treatment success rates because cavitation on radiology is associated with treatment failure and relapse.^25^ Furthermore, 16% to 49% of patients fail to complete antituberculous treatment because of factors such as adverse reactions, cost, and stigma.^26,27,28^ Culture-negative PTB can be treated for a shorter duration.^12,13^ Thus, treatment- and adherence-associated problems might be reduced by the recognition and treatment of early TB.

The viewpoints on how to define culture-negative TB have historically been, and still are, diverse. In settings where it is feasible, the definition of culture-negative pulmonary TB should, if CXR findings are not already consistent with TB, require a chest CT with radiographic abnormalities compatible with TB. In addition, a thorough workup to exclude diagnoses other than TB should be required prior to establishing the diagnosis of culture-negative TB. Given the published data on the lack of diagnostic yield of culture results beyond 3 sputum samples and the ATS and CDC recommendation to obtain 3 sputum samples,^20^ we do not feel it would be justified to propose more than 3 sputum cultures (or NAA assays) or more invasive diagnostic tests such as bronchoalveolar lavage to establish a diagnosis of culture-negative TB, provided that all 3 sputum samples were induced in patients with nonproductive cough and at least 1 sample was obtained in the morning. Nevertheless, we agree with the ATS and CDC recommendations that these approaches should be considered depending on the resources and extent of radiographic abnormalities.

Owing to the documented continuum between asymptomatic *M tuberculosis* infection and early TB disease,^8,11^ it is not possible for us to suggest an acceptable threshold for the proportion of culture-negative TB detection. However, we do feel that the lack of microbiological confirmation in more than 40% of patients diagnosed with PTB globally, as reported for 2017 by the World Health Organization,^1^ is unacceptable and warrants improvement. Based on our prior^4^ and current study results, combined with US and British TB control data,^1,7^ we postulate that among patients without HIV who have PTB, the proportion of culture-negative disease is approximately 15% to 20%. Such early disease, we propose, could remain culture negative even with more aggressive diagnostic approaches because the mycobacteria are more contained in the granulomatous tissue without erosion into the small airways. In fact, detection of 15% to 20% and not a lower proportion could mean improved diagnosis of early TB disease, provided, through thorough workup, that other respiratory diseases are ruled out and the ATS and CDC diagnostic guidelines are followed.

Biomarkers that help identify culture-negative TB are urgently needed. Prior studies assessed the predictive value of interferon γ release assays, but the level of response has not been sufficiently accurate for predicting TB development.^29,30^ A recent prospective study identified blood RNA signatures to predict the risk of TB development and holds promise to be valuable in the detection of very early TB disease.^31^ Such new tools, ideally in the form of simple non-sputum-based biomarker assays that do not require detection of *M tuberculosis* itself, would be extremely valuable as adjunct diagnostics to facilitate the recognition of paucibacillary disease states not detected by current criterion-standard methods.

The observed smaller proportion of men having culture-negative disease in NYC is in accordance with other studies performed in Africa showing that among patients with PTB, men had a higher frequency of radiographic abnormalities, positive results on smear microscopy, and culture positivity compared with women.^32,33,34^ Of note, the recent 2017 increase in NYC TB incidence was associated with an increase in culture-positive cases in males.^6^ Data from both other infections in humans and TB animal models suggest that sex-related immunological differences could, in addition to behavioral reasons, contribute to this observation.^35^ However, whether there could be a possible immunological vs behavioral basis for the association between female sex and culture negativity in TB remains to be explored in prospective studies.

We further note that although nearly all patients with culture-negative PTB were foreign born, with 26% presenting without symptoms, we observed no difference in the proportion of immigrants between the culture-negative and culture-positive groups. While it is conceivable that immigration screening policies for latent TB could have contributed to the detection of some asymptomatic foreign-born patients with culture-negative TB, such detection would likely only occur in recent immigrants who receive TB screening as part of their immigration process.^29,36^ Conclusions about this possibility are not feasible for our study because we did not have information on how long foreign-born patients had lived in the United States by the time of TB diagnosis. Other plausible reasons for the detection of asymptomatic patients with culture-negative PTB include imaging done for unrelated reasons with incidental findings consistent with PTB or more intense screening for TB in general whenever foreign-born patients from TB endemic regions present to health care facilities given their high risk for TB.

### Limitations

This study had limitations. Missing as well as dichotomized data on symptoms limited this study. For example, we did not find a significant difference for the presence of hemoptysis between patients with culture-negative PTB and those with culture-positive PTB. This was surprising because hemoptysis, in general, is a finding associated with cavitary and often more advanced disease, in which case it can be severe.^37^ However, the amount of hemoptysis was not specified in the surveillance data, and thus it could be conceivable that patients with culture-negative PTBs could have had mild hemoptysis due to more trivial causes such as additional bronchitis. As an inherent limitation of analyzing a surveillance database, we could determine neither the severity of hemoptysis nor the reasons why hemoptysis was recorded in patients with culture-negative PTB. We further note that a significantly lower proportion of patients with culture-negative PTB reported hemoptysis compared with those who had culture-positive PTB when missing data were coded as not present instead of excluded from analysis (13% vs 23%; *P* = .02) (Table 3). Similarly, differences between the groups became also significant for sputum production, fever, and night sweats. This analysis approach likely reflects the true proportion of symptoms present in each group because clinicians do not regularly document all negative findings. Missing data further limited our analysis and conclusions for the comparison of symptom duration.

Another limitation was that we could not be certain that all patients with culture-negative PTB without later *M tuberculosis* confirmation had TB. Nonetheless, these patients were diagnosed by a licensed clinician, had their cases verified by a NYC health department case manager, and had their data further reviewed using the CDC’s diagnostic criteria.^5,13,14^ Furthermore, patients with culture-negative PTB were treated with antituberculous therapy with consequent clinical and/or radiographic improvement; therefore, we felt confident about their TB diagnosis. Using similar criteria, a recent noninferiority trial comparing a 4-month rifampin regimen vs a 9-month isoniazid regimen for the treatment of latent TB infection also used clinically diagnosed TB without microbiological evidence as a secondary outcome.^38^ This emphasizes that TB experts consider clinically diagnosed or nonmicrobiologically confirmed TB an important disease entity worth acknowledging. Further acknowledgment is provided by the ATS, CDC, and Infectious Diseases Society of America, who suggest a 4-month treatment regimen for patients with culture-negative PTB.^17^ Another limitation was that different TB experts define “culture-negative” TB differently. Because some patients in our database had additional workup that others did not receive, and because we needed to achieve consistency across patients’ enrollment and data interpretation, we restricted defining sputum culture results based on the first 3 sputum samples as per official guidelines by the ATS and the CDC.^20^ We note that it is standard of care for clinicians in NYC to order sputum samples induced with hypertonic saline to collect adequate samples, particularly for those with nonproductive cough. We further note that categorizing patients with culture-negative PTB with a later culture-positive sample as having culture-positive PTB did not change statistical significance when comparing the groups.

## Conclusions

In conclusion, our NYC population-wide data showed that approximately 15%, a considerable proportion, of all patients without HIV who had PTB had sputum culture-negative results. They presented with fewer symptoms and less cavitation on radiologic imaging compared with patients with culture-positive PTB. An increased awareness of the lower probability of clinical signs and manifestations of this very early disease state, ideally combined with consulting a TB specialist and performing a chest CT scan, could enhance the recognition of culture-negative PTB. Such enhanced recognition could improve timely antituberculous treatment initiation and prevent the development of transmissible disease. Our findings further emphasize the continuous need for better non-sputum-based biomarkers to help detect early states of TB. Local health departments should consider closely following high-risk patients with potential culture-negative PTB if left untreated and should recommend adding additional accurate non-sputum-based biomarker tests should these become available.
